# Digital Therapeutics: Exploring the Possibilities of Digital Intervention for Myopia

**DOI:** 10.3389/fdgth.2021.710644

**Published:** 2021-08-27

**Authors:** Yong Sun Lee, Seung Eun Choi, Jarang Hahm, Myoung Joon Kim, Hyo Sook Bae, Kayoung Yi, Hyun Taek Lim, Joon Young Hyon

**Affiliations:** ^1^S-Alpha Therapeutics, Inc., Seoul, South Korea; ^2^Renew Seoul Eye Center, Seoul, South Korea; ^3^Black Kactus LLC., Seattle, WA, United States; ^4^Department of Ophthalmology, Hallym University Kangnam Sacred Heart Hospital, Seoul, South Korea; ^5^Department of Ophthalmology, Asan Medical Center, College of Medicine, University of Ulsan, Seoul, South Korea; ^6^Department of Ophthalmology, Seoul National University College of Medicine, Seoul University Bundang Hospital, Seongnam, South Korea

**Keywords:** digital therapeutics, pediatric myopia, software algorithm, software as medical device, dopamine, IGF-1, cortisol, neuronal-humoral factors

## Abstract

Pediatric myopia is increasing globally and has become a major public health issue. However, the mechanism of pediatric myopia is still poorly understood, and there is no effective treatment to prevent its progression. Based on results from animal and clinical studies, certain neuronal–humoral factors (NHFs), such as IGF-1, dopamine, and cortisol may be involved in the progression of pediatric myopia. Digital therapeutics uses evidence-based software as therapeutic interventions and it has the potential to offer innovative treatment strategies for pediatric myopia beyond conventional treatment methods. In this perspective article, we introduce digital therapeutics SAT-001, a software algorithm that modulates the level of NHFs to reduce the progression of pediatric myopia. The proposed mechanism is based on a theoretical hypothesis derived from scientific research and clinical studies and will be further confirmed by evidence generated from clinical studies involving pediatric myopia.

## Introduction

Myopia is one of the leading eye disorders that causes significant visual impairment. Flitcroft et al. (1) defined and classified myopia as axial and refractive myopia based on its type and severity. Axial myopia is often associated with excessive axial lengthening (axial length > 25.5 or 26.5 mm), whereas refractive myopia can result from changes in the anatomy or the location of image-forming components, such as the cornea and the lens (1). In axial myopia, vision begins to deteriorate as the axial length of the eye becomes greater than normal; this elongation begins in childhood and adolescence, with the largest growth found in patients between 8 and 15 years of age (2). High myopia is a condition with an axial length >26–27 mm, which can result in loss of vision due to retinal detachment, neovascularization, cataract, glaucoma, or macular atrophy (3).

The increasing prevalence of this condition, referred to as “myopia boom” by some researchers (4), affects every region of the world differently. In the United States, myopia affects 33% of adult individuals (5), and ~4% of affected people have high myopia, which is associated with an increased risk of serious ocular complications that leads to vision loss (6). In 2020, the highest estimates worldwide occurred in East Asia (51.6%) and Southeast Asia (46.1%). These two regions will continue to bear the largest burden by 2050 (East Asia, 65.3%, Southeast Asia, 62%) (7). In Singapore and Taiwan, the prevalence of myopia in young adults between 12 and 18 years of age was 85–90% (5). In Korea, a survey of 33,922 patients conducted from 2008 to 2012 showed that the age-standardized prevalence of myopia (SE ≤ −0.75 D) and high myopia (where SE < −6.0 D) in this age group was 80.2 and 9.3%, respectively (8).

The rapid increase in the prevalence of myopia suggests that myopia is a multifactorial condition, in which the interplay of genetic and environmental factors contributes to myopia development (9). Education and urbanization (10), time spent outdoors (11, 12), wavelength of light (13), and physical activity (14) are examples of environmental factors related to myopia progression.

Recent reviews extensively covered the progress of molecular biology technologies such as linkage analysis, genome-wide association studies, and next-generation sequencing (NGS)-based exome sequencing in myopia genetic analysis. A total of 25 myopia loci were identified by genome-wide linkage analyses, among which eight loci were replicated in multiple ethnic groups and four were causal genes (15, 16). Positive association to myopia was found in genes involved in extracellular matrix (ECM) growth and remodeling (17). Studies using NGS identified 17 causal genes for myopia (15, 16).

Progressive myopia is a serious ocular disorder that can be irreversible once the progression has started (2), and affects the quality of life in children. Currently, there is no effective therapeutic intervention for preventing myopia in children and adolescence. The lack of effective treatment options for myopia is due to limited knowledge about the genetic contribution of variants in myopia and the interaction of genes with environmental factors. Therefore, there is an urgent need for effective intervention to control the progression of myopia that is minimally invasive to children.

## Current Interventions

Initially, bifocal spectacles (or multifocals) were used to control myopia progression, but they showed no significant effect on slowing myopia (18, 19). Recently, however, studies using peripheral defocus ophthalmic lens (DIMS spectacle lenses) slowed the progression in children with myopia (20).

MiSight Contact Lens (CooperVision) is the first lens approved by the U.S. Food and Drug Administration (FDA) to control the progression of myopia in children (21). Randomized clinical trial (RCT) results showed that MiSight is safe and effective in slowing the progression of myopia over 2 (21) to 3 years (22). Orthokeratology (Ortho-K), approved by the FDA in May of 2021, are a series of hard, hydrophilic, gas-permeable contact lenses designed to gradually flatten the central cornea. Ortho-K suppressed axial elongation and slowed down the progression of myopia in children by up to 45% (2). Despite these promising findings, Ortho-K does not permanently stop the progression of myopia. It carries a serious risk of microbial keratitis, and the rebound has also been observed in patients who discontinue this form of treatment (2).

Atropine is the sole prescription drug that can control the progression of myopia, but the precise mechanism of action (MOA) remains unknown (23). Studies have shown that inhibition of myopia progression is dose-dependent, and a stronger concentration of the eyedrops showed a higher effect (24, 25). Rebound may or may not occur depending on the dose. In one study, no rebound effect was observed within the first year after discontinuing low-dose atropine; in another, myopia returned to the baseline after discontinuing 1% atropine (2). Long-term safety is also not fully understood, and short-term photophobia has been observed in patients who used 1 or 0.5% atropine (26).

**Figure 1:**
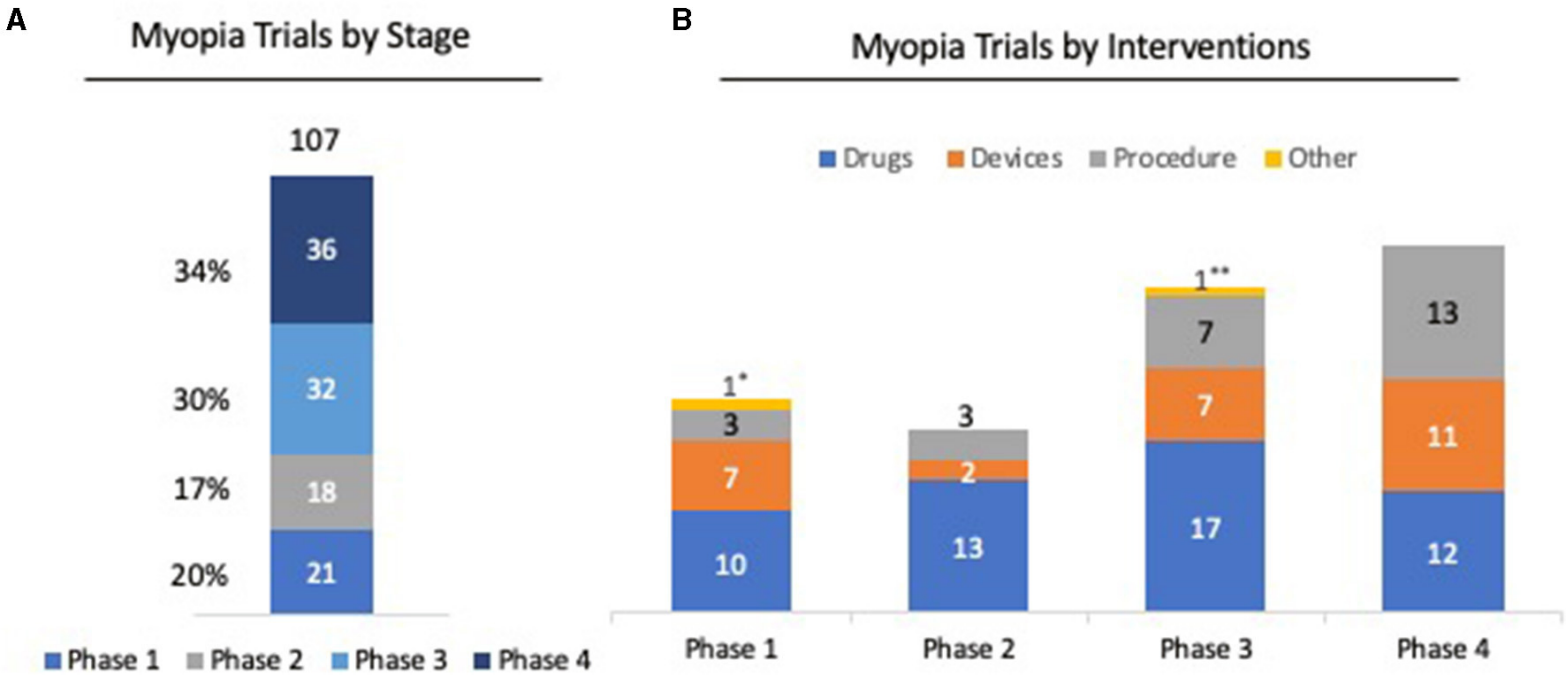
Clinical trial trends in myopia. **(A)** Among 107 clinical trials registered for myopia, 36 (34%) of clinical trials have entered or completed Phase 4; 32 (30%) are in Phase 3; 11 (7%) are in Phase 2; and 21 (20%) are in Phase 1 stage. **(B)** Analysis of clinical phases by intervention shows that 52 trials (48%) were for drugs for myopia, 27 trials (25%) were on devices, and 26 trials (4%) were on procedures, such as LASIK and SMILE. ^*^Clinical trial on dietary supplement for myopia. ^**^ Clinical trial on family-based outdoor program for myopia. Source: ClinicalTrials.gov. Keyword: Myopia; Phase 1-4; status; active but not recruiting/recruiting/not yet recruiting/completed Accessed Feb 2021.

Evidence shows that there is a more rapid progression of myopia in children who have onset at younger ages (27). Progressive myopia can be irreversible and increases the risk of developing into high myopia, which is associated with serious ocular diseases. Results showed that slowing myopia progression in children by one diopter could lower the risk of developing maculopathy by 40% (28). Currently, there are more than 100 clinical trials, from early to late phase, studying the safety and efficacy of various drugs, devices, and procedures to slow down the progression of myopia (Figure 1). Finding effective therapies for slowing the progression of myopia in children is critical in reducing the prevalence of myopia around the globe.

## Novel Digital Therapeutics for Pediatric Myopia

As an emerging innovative treatment approach, digital therapeutics is being quickly adopted in the clinical field. Current digital therapeutics involves the use of a smartphone or iPad application (“app”)-to deliver unique software-based clinical therapies through a digital interface, or software used in combination with a drug prescription (29). In digital therapeutics, proprietary algorithms are responsible for the mode of action as well as the accumulation, generation, and output of health data (30). Advances in myopia research indicate a strong interplay between genetic factors and environmental factors in myopia development (31). This suggests that genetic components, as well as psychological, neuronal, and humoral factors, may have an important role in myopia progression.

**Figure 2:**
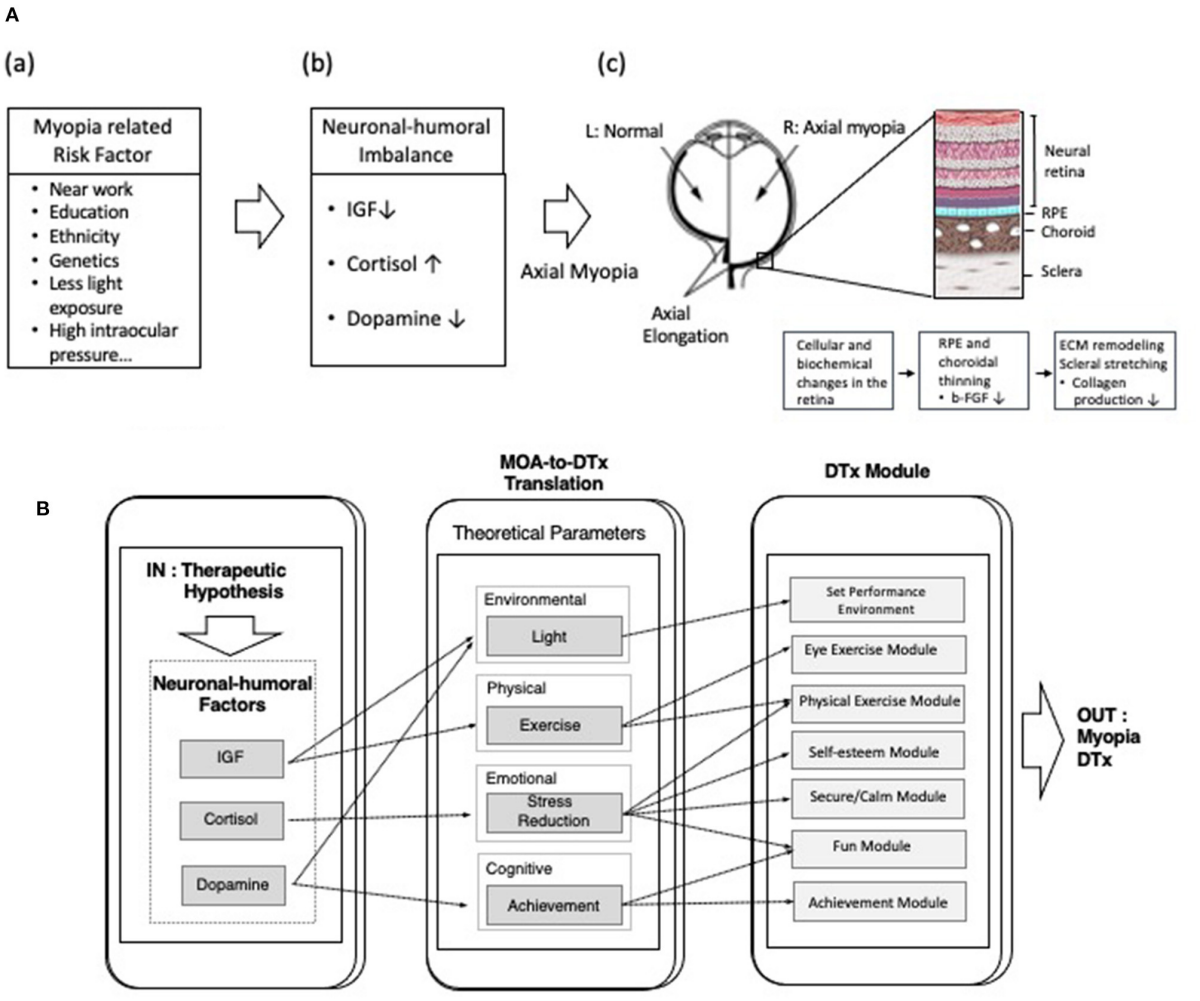
**(A)** Schematic illustration of the hypothesis that imbalance in the level of neuronal–humoral factors (NHFs) affects axial elongation in pediatric myopia. (a) Various risk factors (near work, education, ethnicity, genetics, light exposure, and high intraocular pressure etc.) are known to cause myopia. (b) Imbalance of NHFs such as insulin-like growth factor (IGF), cortisol, or dopamine may affect the progression of axial myopia. (c) In axial myopia, vision begins to deteriorate as the axial length of the eye becomes greater than normal (axial elongation). During axial elongation, cellular and biochemical changes occur in eye's posterior components, such as thinning of retinal pigment epithelium, choroid, and sclera. Disruption of cross linking of fibrils and fibers and reduction of the transverse cross-linking of collagen network in the sclera, leads to pathological stretching of choroid and sclera. **(B)** Schematic diagram of showing the level of neuronal–humoral factors regulated by corresponding theoretical parameters (environmental, physical, emotional, cognitive parameters), which are then translated into digital instruction. The outcome is novel digital therapeutics (DTx) for myopia.

These findings led to the development of novel digital therapeutics for pediatric myopia (Figure 2A). S-Alpha Therapeutic's SAT-001, a proprietary software algorithm, aims to delay the progression of pediatric myopia by modulating the balance of neuronal–humoral factors (NHFs) related to myopia progression.

The therapeutic hypothesis of SAT-001 is to modulate the balance of NHFs related in myopia by controlling corresponding theoretical parameters. These NHFs were deduced by performing meta-analysis and data mining on scientific publications and clinical trial results on axial myopia in children and adolescents. Theoretical parameters are classified as: environmental (light), physical (exercise), emotional (stress reduction), and cognitive (a sense of achievement) (Figure 2B). A digital therapeutics module converts each theoretical parameter into specific digital instructions (input) delivered through an application and executed by the patient (output). As examples, the digital therapeutics module for the physical parameter provides instruction on specific tasks, such as eye exercises and low-intensity daily exercises; the emotional parameter guides the patient to feel relaxed, comfortable, safe, satisfied, entertained, and accomplished.

## Factors Involved in Myopia Digital Therapeutics

### Growth Factors

Therapeutic intervention of SAT-001 delays the progression of pediatric myopia by using controlled eye exercises that modulate the level of certain NHFs.

The first NHF is the neurotrophic growth factor (NGF). NGFs affect the synthesis of proteoglycan and glycoprotein that strengthen the ECM structuring of the sclera (32). The pathological changes that occur in a myopic eye deform and stretches the sclera membrane to such a degree that the choroid and sclera suffer irreversible vision decline (33, 34).

Specific growth factors have been shown to affect scleral proteoglycan synthesis in ECM remodeling (35). One example is the insulin-like growth factor 1 (IGF-1) (36), an NGF which is known to play a role in muscle growth during development and regeneration (37, 38), that increased during resistant exercise (39, 40). Animal studies indicate the protective role of IGF-1 in maintaining the natural shape and size of the eyeball by enhancing the strength of ECM. IGF-1 protected the growth of retinal endothelial cells in oxygen-induced retinopathy in mice (41) and the ocular growth in chickens (42).

Genetic association studies showed that single nucleotide polymorphism observed in IGF-1 was significantly associated with different types of myopia, including high myopia in Caucasian and Chinese populations (43, 44).

Reports on the effect of eye exercises on myopia development (45–47) suggest that eye motion contributes to the development of emmetropization, which occurs when the optical axis length adjusts to ensure that the retina is properly placed. Studies have shown that abnormal oculomotor behavior may contribute to the development of myopia (48). Mechano-growth factor (MGF), a splice variant of IGF-1, is synthesized and secreted when extraocular muscles are used in exercise (49). Extraocular muscles are striated muscles that originate from the episcleral shell directly and control eyeball movement voluntarily. The IGF-1 as MGF is synthesized and secreted when the voluntary contraction of extraocular muscles occurs by eyeball movements.

Together, these studies suggest the important role of IGF-1 in ocular growth and in the progression of myopia. Growth factors such as IGF-1 released by eye exercise were selected as one of the key NHFs for SAT-001 to control the progression of myopia.

### Retinal Dopamine

The second NHF associated with SAT-001 is dopamine. Dopamine is a key neurotransmitter in the brain and retina that supports motor, cognitive, and visual functions.

Since the first report on the role of retinal dopamine in suppressing myopia (50), extensive studies have been published on the involvement of dopamine in myopia development (51). In the form-deprivation myopia chicken model, low dopamine levels extended axial growth of the eye, whereas high dopamine levels suppressed abnormal axial growth (50). Studies using apomorphine, a non-specific dopamine agonist, reduced the amount of induced myopia in both chicken and monkey models (52, 53).

Light stimulation increases retinal dopamine in dopaminergic amacrine cells by the response of rods, cones, and intrinsically photosensitive retinal ganglion cells (ipRGCs) to light (54). The protective effect of bright light was first reported in several animal studies. In chickens, exposure to high illumination levels (15,000 lux) for 5 h a day significantly reduced the development of form-deprived myopia, when compared with chickens reared with translucent diffusers under normal laboratory lighting levels (500 lux) (55). In rhesus monkeys, an inhibitory effect was observed on form-deprived myopia, when reared for 6 h a day in high ambient light (25,000 lux) (56).

Growing evidence suggests that retinal dopamine stimulated by a bright light in outdoor activity has a protective effect against myopia progression. Several epidemiological studies have reported on the inverse correlation between outdoor activity and myopia development (57). Jones et al. (58) noted that children who spent 15 h/week (2.1 h per day) outdoors had one third the risk of becoming myopic, compared with children who spent <5 h per week (0.7 h per day) outdoors. Similar results were found in a report of metaanalysis that included seven crosssectional studies. Sherwin et al. (11) analyzed the time spent outdoors and myopic progression and found consistent evidence that increasing the time spent outdoors by an additional hour per week reduced myopia progression by 2%.

Recently, a study stimulating the blind-spots in myopic eyes with blue-light was reported (59). Fifteen healthy non-myopic and myopic young adults underwent stimulation with blue light using virtual reality (VR) headset devices. The level of retinal activity related to the dopamine pathway was increased in myopic eyes after blue light stimulation. This supports previous findings that exposure to daylight (rich in blue light) during outdoor activities delays the onset of myopia and involves the dopaminergic system. This was the first study evaluating the effect of stimulating the myopia eye using a VR digital therapeutics device.

Based on these findings, SAT-001 incorporated light parameters to modulate the light environment for therapeutic intervention. The potential benefit gained by using SAT-001 is to induce retinal dopamine release and delay the progression of myopia through daily light exposure and physical activity.

### Stress and Cortisol

The third NHF incorporated in SAT-001 is cortisol, a glucocorticoid that is secreted in response to stress in the human adrenal cortex. Studies have shown that psychological stress may be one of the major causes of visual system diseases, such as glaucoma, optic neuropathy, and myopia (60, 61). In a study involving 87 subjects with high myopia (<-10 D), the level of serum cortisol and other steroid hormones was significantly higher in the high myopia group compared with the control group (62). In recent studies, ocular diurnal–circadian rhythms synchronized to a 24-h solar day, implicated in myopia, were examined in children from 5 to 14 years of age (63). Ocular parameters in diurnal rhythms such as corneal thickness, axial length, intraocular pressure, choroidal thickness, including the cortisol level were measured every 4 h in a 24-h period. Cortisol maintained low concentration during the night, but peaked in the morning, consistent with previous studies (64). Axial length was the longest in the morning and then decreased throughout the evening, whereas choroid, the vascular structure of the outer retina, was approximately antiphase with axial length. These results show that the cortisol rhythms are in phase with axial length and provide evidence that cortisol could potentially influence myopia progression. Studies show that an increased level of cortisol inhibits collagen synthesis in connective tissues and isolated fibroblasts (65). Shifts in hormone levels cause deregulation between matrix metalloproteinases and tissue inhibitors of metalloproteinases (TIMs), which disrupt cross linking of fibrils and fibers and reduce the transverse crosslinking of the collagen network in the sclera (66), leading to sclera degradation [Figure 2A(c)].

To induce relaxation response and reduce the level of stress hormones in patients, an emotional parameter is included as the therapeutic element of SAT-001. The role of deep breathing in reducing stress and lowering the level of cortisol has also been reported in clinical studies (67). In some cases, improved breathing also increases the secretion of growth hormones, including IGF-1, without cortisol surge (68). Reduction of cortisol could prevent the inhibitory effect of stress hormone in decreasing sclera tension during myopia deterioration.

## Discussion

Like most emerging digital health technologies, digital therapeutics for pediatric myopia is relatively new and faces several specific challenges.

First, like traditional drug and medical device development, rigorous clinical trials must prove the clinical value of digital therapeutics in treating pediatric myopia. SAT-001 is in the early proof-of-concept stage. Although there are research publications supporting the role of NHFs on myopia progression, clinical evidence is not yet available. For digital therapeutics to be fully utilized in pediatric myopia, the technology needs to be validated in clinical trials and approved within regulatory frameworks. To address this issue, a proof-of-concept clinical study will be conducted on a limited number of pediatric myopia patients under controlled conditions to obtain preliminary data on the efficacy and safety of SAT-001. Based on these results, a RCT will be performed to generate the clinical evidence necessary for approval by regulatory authorities.

Second, digital therapeutic products present unique challenges for regulatory authorities. Digital therapeutics is software integrated into digital platforms to serve medical purposes. Digital therapeutics applications that make claims about the diagnosis or treatment of the disease are subject to FDA regulation. However, traditional drug-quality measures do not apply to digital therapeutics, which rely on software that will continuously evolve beyond the initial approval. To meet this challenge, the FDA launched the Digital Health Software Precertification Program, a pilot program to expedite the regulatory approval for Software as Medical Device (SaMD) products (69). These guidelines reflect the efforts of the FDA to make more universal, consistent rules for software classified as SaMD. For digital therapeutics manufacturer seeking regulatory approval, SaMD products should have various recommendations attached to the software for analytical purposes and outline any potential adverse consequences. The software should be fully documented to identify its role and its place within the clinical environment. The unique regulatory requirement of SaMD products must be understood and the most effective quality management systems must be implemented from the early stage of product development.

Third, patient engagement and motivation can be challenging in digital therapeutics. Digital therapeutics can use different patient-facing digital forms, such as smart phone applications, video games, and virtual reality. Video games as a clinical intervention have demonstrated the benefits of improving vision in healthy individuals, as well as those with amblyopia (70, 71).

Digital therapeutics using video games improved vision acuity and stereopsis in both amblyopic children and adults (72, 73). Birch et al. reported on improved visual acuity in preschool children by repeated binocular visual treatment with dichoptic iPad games. Visual acuity with binocular iPad game play improved over a 4-week period, and children who played for 8 h over a 4-week period had significantly more improved visual acuity compared with children who played for up to 4 h (74).

SAT-001 also uses a video game-like interface, which helps children feel comfortable with the digital device and increases their attention while executing the assigned instructions. A reward system is incorporated to motivate children to complete their assigned sessions and to increase self-confidence. Children enrolled in clinical studies range widely in age: from preschool (5–7 years old) to older school-age children (8–12 years old). Designing different levels of video-game interventions appropriate for each age group to ensure engagement and high adherence to digital therapeutics is critical.

Despite the challenges, digital therapeutics products are generally easy for patients to use and they decrease the cost of treatment. Having access to therapeutic intervention on personal mobile devices will allow these young patients the freedom to receive treatment outside the confines traditionally imposed when meeting with a clinician. A web-based platform allows clinicians to access and monitor the clinical data of a patient and follow their treatment progress in real time, enabling them to identify any issues with patient compliance and customize their therapy.

## Conclusions

Innovation through the convergence of pharmaceuticals, medical devices, and software led to the advancement of digital therapeutics. In the field of myopia, ongoing research is providing some insights into the biochemical molecules and neurotransmitters underlying the progression of myopia. Digital therapeutics SAT-001 aims to delay the progression of pediatric myopia through proprietary software that modulates the balance of NHFs implicated in myopia. The proposed mechanism is based on a theoretical hypothesis derived from meta-analysis of data from research publications and clinical studies. This hypothesis will be further confirmed by evidence generated from clinical studies involving pediatric myopia.

Even though many digital therapeutics products and technologies are in the early stages, the increase in research and development efforts, and the results gained through clinical evidence, could provide promising opportunities to treat diseases with high unmet medical needs.

## Data Availability Statement

The original contributions presented in the study are included in the article/supplementary material, further inquiries can be directed to the corresponding author/s.

## Author Contributions

YL, SC, MK, and JH contributed to the invention of this manuscript. HB, HL, KY, and JYH reviewed the manuscript. All authors contributed to the article and approved the submitted version.

## Conflict of Interest

YL, SC, and MK are stockholders of S-Alpha Therapeutics. YL, SC, and JH are employed by S-Alpha Therapeutics. HB is employed by Black Kactus, LLC. HB received consulting fees from S-Alpha Therapeutics. The remaining authors declare that there is no commercial or financial relationship in the research conducted that could be constructed as a potential conflict of interest.

## Publisher's Note

All claims expressed in this article are solely those of the authors and do not necessarily represent those of their affiliated organizations, or those of the publisher, the editors and the reviewers. Any product that may be evaluated in this article, or claim that may be made by its manufacturer, is not guaranteed or endorsed by the publisher.
